# Iron and Manganese Retention of Juvenile Zebrafish (*Danio rerio*) Exposed to Contaminated Dietary Zooplankton (*Daphnia pulex*)—a Model Experiment

**DOI:** 10.1007/s12011-020-02190-z

**Published:** 2020-05-23

**Authors:** Petra Herman, Milán Fehér, Áron Molnár, Sándor Harangi, Zsófi Sajtos, László Stündl, István Fábián, Edina Baranyai

**Affiliations:** 1 Department of Inorganic and Analytical Chemistry, Atomic Spectroscopy Partner Laboratory, University of Debrecen, Debrecen, H-4010 Hungary; 2 Faculty of the Agricultural and Food Sciences and Environmental Management, University of Debrecen, Debrecen, H-4032 Hungary

**Keywords:** Trophic transfer, Iron, Manganese, Elemental analysis, *Daphnia pulex*, *Danio rerio*

## Abstract

In present study the effect of iron (Fe) and manganese (Mn) contamination was assessed by modeling a freshwater food web of water, zooplankton (*Daphnia pulex*), and zebrafish (*Danio rerio*) under laboratory conditions. Metals were added to the rearing media of *D. pulex*, and enriched zooplankton was fed to zebrafish in a feeding trial. The elemental analysis of rearing water, zooplankton, and fish revealed significant difference in the treatments compared to the control. In *D. pulex* the Mn level increased almost in parallel with the dose of supplementation, as well as the Fe level differed statistically. A negative influence of the supplementation on the fish growth was observed: specific growth rate (SGR%) and weight gain (WG) decreased in Fe and Mn containing treatments. The redundancy analysis (RDA) of concentration data showed strong correlation between the rearing water and *D. pulex*, as well as the prey organism of Fe- and Mn-enriched *D. pulex* and the predator organism of *D. rerio*. The bioconcentration factors (BCF) calculated for water to zooplankton further proved the relationship between the Fe and Mn dosage applied in the treatments and measured in *D. pulex*. Trophic transfer factor (TTF) results also indicate that significant retention of the metals occurred in *D. rerio* individuals, however, in a much lower extent than in the water to zooplankton stage. Our study suggests that Fe and Mn significantly accumulate in the lower part of the trophic chain and retention is effective through the digestive track of zebrafish, yet no biomagnification occurs.

**Figure:**
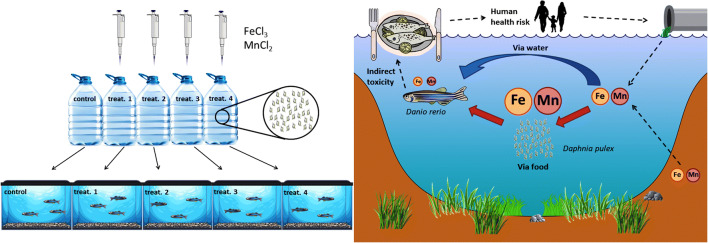
Graphical abstract

Graphical abstract

## Introduction

Aquatic ecosystems are considered to be the most sensitive environmental media. Emitted pollutants tend to enter first to surface waters, and through the aquatic food chain almost all living organisms can be affected. Metal pollution of surface waters is an ever increasing environmental issue of the last few decades. Pollutants are released into the aquatic ecosystems via anthropogenic activities (industrial and agricultural production, traffic, untreated wastewater effluent etc.); however, the natural geological background can also result in a higher concentration of elemental impurities in the water body [1–3]. Metals accessing to water are usually the lack of biodegradability compared to some organic materials that can be metabolized into less harmful substances [2]. Beside the direct adverse effect of the inorganic contamination on the aquatic plants and organisms resulting in abnormalities, metals can be accumulated in the cells and organs of living individuals [4–6]. Since the trophic levels setting up the aquatic ecosystem are strictly built on each other, more advanced and more complex organisms consuming species from the lower aquatic taxonomy class can create much higher concentrations in their tissue trough bioaccumulation than it was in the initial environmental media [7]. Bioconcentration thus affects nearly all the living organisms in the aquatic ecosystem as well as the human food chain [4, 5], which keeps the subject highly significant [3].

There are several factors on which the tendency and level of bioaccumulation depend: from the distribution of the contaminants through the physical and chemical circumstances (such as salinity, redox conditions, total suspended solid concentration) to the biology of the affected living organisms (feeding strategy, age, assimilation efficiency, etc.) [8, 9]. Zhou et al. in their review considering the assessment of metal pollution of aquatic ecosystems concluded that in order to gain a deeper understanding of the pollution routes in aquatic media, further research of bioavailability is necessary [10]. The condition of water ecosystems can be monitored by the continuous analysis of physical and chemical variables; however, significant additional information can be gained by modeling water-related pollution and investigating the effects on different trophic levels by indicator organisms. Biomonitoring plays an important role in this approach offering bioaccumulation level and toxicological effects to support the comprehensive assessment [11]. Toxicity tests are carried out to investigate the effects of various chemicals and contaminants on living organisms, comparing the sensitivity of the different species [12–14]. They are most commonly applied to examine the effect of the polluted media on the vitality, agility, and survival of the indicator organisms [15]. However, by bioindicators, it is also possible to investigate the accumulation of certain pollutants along the food chain, thus gain more detailed information regarding water quality [16–18]. In order to examine the complex effect of more than one contaminant and to evaluate the health condition of the entire aquatic ecosystem, it is important to consider the enrichment along the trophic chain and to examine the biomagnification in the food webs as well. Relationships within the life community can be modeled by multispecies toxicity tests involving more taxa, which are built on each other in the trophic system [19–22].

Both in active and passive aquatic toxicity studies bacteria, algae, zooplankton, and fish species are most commonly examined [23]. These organisms represent different trophic levels in the food chain and—depending on their sensitivity—can provide information about the extent of the pollution in surface waters. At the level of zooplankton organisms, *Daphnia pulex* is one of the most common bioindicator species of freshwaters due to the many characteristics facilitating its application in biochemical and toxicological studies as well as its relatively simple and cheap raising under laboratory conditions [14, 15, 24]. *Daphnia* species are considered to be excellent indicators of the surrounding water, and being at the base of the food chain, it is serving as resource for consumers on higher trophic levels, including fish. Zebrafish (*Danio rerio*) is on the next level in the trophic system and also a very commonly used indicator organism [25, 26]. Due to its small size, rapid growth, ease of access, and other favorable biological features, individuals are often used not only in toxicity tests but also in biomedical trials. Fernández et al. mentioned in their very recent article that a steady increase can be observed in the past 15 years of publications containing “zebrafish” and “toxic” in the title, reaching an equal to that of the mouse [27].

It is important to take into account the possible routes of exposition when investigating the toxic effect of a chemical. Depending on the form of the metal compound, uptake paths are through the permeable epidermis or via food ingestion. Since the entire body surface of the aquatic organism gets in connection with the contaminants the adverse effects on the outer epidermis, the digestive tract and the respiratory system may add [28].

According to Koivisto [29] the development of complex test systems corresponding to real nature is needed to better assess and monitor the aquatic environment, while Zhou et al. suggested the establishment of a precaution system for metal pollution including biomonitoring network. In the scope of these approaches the deeper understanding of the metal retention through the food chain is important and can be supported by model experiments under laboratory conditions [22, 30, 31]. Field studies alone are not enough to distinguish between dietary and water-based metal retention [32].

Several studies can be found in the scientific literature investigating the toxic metal retention along the aquatic food web; however, most of them are field studies of freshwater ecosystems [33–37] and even more of them describe results for marine environment [38–40]. Considering the analyzed element, less data are available about Fe and Mn compared to toxic metals such as Pb, Cd, As, or Hg [32, 41–44]. Accordingly, toxic metal concentrations are more strictly regulated. WHO standards for drinking water (2011) has no strict guideline for either Fe or Mn [45] (previous guidelines were discontinued in the latest editions), while USEPA (2012, EPA 822-S-12-001) states maximum levels of 50 μg L^−1^ and 300 μg L^−1^, respectively. EC (1998/83/E) directive is the same for Fe and 200 μg L^−1^ for Mn. Regarding the surface waters and the level of protection necessary for aquatic life, water quality standards give no acute criteria for Fe but contain chronic criteria of 1.000 μg L^−1^. Considering Mn, neither acute nor chronic criteria are stated.

Thus, the aim of the current study is to investigate the trophic transfer and biomagnification of Fe and Mn along the aquatic food chain by exposing the metals to juvenile zebrafish via dietary *Daphnia pulex*. Both metals are important micronutrients and essential for aquatic organisms until reaching a certain concentration limit. The Fe level was reported to show a continuously increasing pattern in Swedish and Finnish surface waters over the last 10 years [46], as well as Mn is considered to be an emerging contaminant in the aquatic environment [47]. The Fe and Mn level of the Hungarian oxbows in the Upper-Tisza region is recently reported to be high as a result of our previous assessment [48] since when we started to conduct model experiments involving these elements. Based on the pollution index of sediment samples, the studied oxbows were characterized by moderate levels of contamination for Fe and Mn, since the mean geochemical concentration in the upper level (0–10 cm) of floodplain sediments is exceeded for both elements (35.000 mg kg^−1^ for Fe_2_O_3_ and 1000 mg kg^−1^ for MnO) [49] [50]. In our first study the Fe and Mn retention was investigated via the rearing water media [28], while in this paper the results of accumulation tendency through food ingestion is described. The elemental concentration of the indicator organisms was determined by microwave plasma atomic emission spectrometry (MP-AES), which is a new and cost-effective technique that can be adapted in the routine analysis of metal pollution.

## Materials and Methods

### Animals

Animal handling and experimental procedures followed the Directive 2010/63/EU of the European Parliament and of the council on the protection of animals used for scientific purposes (2010/63/EU, Official Journal of the European Union. L 276/33. 20.10.2010.)

Water conditions were the same for both the maintenance and treatment and checked regularly. Parameters among the aquaria were not significantly different (*p* > 0.05) and did not affect the treatments. Dissolved oxygen (DO), temperature, and pH were tested daily by HACH LANGE HQ30D. The level of ammonia, nitrite, and nitrate were measured weekly by spectrophotometry (HACH LANGE DR3900) according to the related USEPA methods (NH_4_-N: USEPA NESSLER METHOD, NO_2_-N: USEPA DIAZOTIZATION METHOD, NO_3_-N: CADMIUM REDUCTION METHOD).

During the experimental period, the following average water quality parameters were determined:

$$ \mathrm{DO}:7.55\pm 0.34\ \mathrm{mg}\ {\mathrm{L}}^{-1} $$

$$ \mathrm{Water}\ \mathrm{temperature}:22.65\pm 0.91{}^{\circ}\mathrm{C} $$

$$ \mathrm{pH}:8.61\pm 0.09 $$

$$ {{\mathrm{NH}}_3}^{+}:0.18\pm 0.07\ \mathrm{mg}\ {\mathrm{L}}^{-1} $$

$$ {{\mathrm{NO}}_2}^{-}:0.07\pm 0.11\ \mathrm{mg}\ {\mathrm{L}}^{-1} $$

$$ {{\mathrm{NO}}_3}^{-}:12.6\pm 6.1\ \mathrm{mg}\ {\mathrm{L}}^{-1} $$

### Enrichment of *Daphnia pulex* with Fe and Mn

*Daphnia pulex* individuals were originally collected from a pond then reared isolated and enriched under laboratory conditions in a model system, which ensured optimal environmental conditions for the culture of the zooplankton organisms. The consisting 4-L volume plastic tanks were filled up with continuously aerated tap water, which temperature was kept at 22 °C and a 16–8 h (light-dark) of illumination was provided. A day prior to dietary serving the zooplankton, 0.6 g wet mass of *Daphnia* was measured into each of the fifteen plastic containers and enriched for 24 h according to the following treatments:

1. Fe: 5.70 mg L^−1^ + Mn: 2.90 mg L^−1^
2. Fe: 5.70 mg L^−1^ + Mn: 6.25 mg L^−1^
3. Fe: 15.0 mg L^−1^ + Mn: 2.90 mg L^−1^
4. Fe: 15.0 mg L^−1^ + Mn: 6.25 mg L^−1^

control, no supplementation

Concentrations were adjusted considering our preliminary study where the retention was investigated via rearing water [28]. In this work ten times the previously applied sublethal level of Fe and Mn was adjusted to investigate the potential accumulation effect of a toxic concentration range via the chosen aquatic food chain.

The solutions of solid FeCl_3_ and MnCl_2_ (analytical purity, SPEKTRUM 3D) were used to adjust the aforementioned concentrations in the model media. Control treatment contained only tap water with the elemental content of the following: Cu: 7.0 μg L^−1^, Fe: 5.0 μg L^−1^, K: 2.70 mg L^−1^, Mg: 16.1 mg L^−1^, Mn: 2.0 μg L^−1^, Na: 31.6 mg L^−1^, Sr: 0.40 mg L^−1^, and Zn: 41 μg L^−1^, according to ICP-OES analysis. Each treatment was set in triplicate (*n* = 3), and the plastic containers were arranged in a completely randomized design. After the enrichment period, the harvested *D. pulex* organisms were filtered by plankton net of 150-μm mesh size and rinsed with ultrapure water (Millipore MilliQ) in three times to evade contamination of the rearing media. *Daphnia pulex* was fed ad libitum to the zebrafish juveniles.

### Enrichment of *Danio rerio* with Fe- and Mn-Contaminated Daphnia

Zebrafish juveniles of 60 dph were purchased from a local fish market, and a 48 h of acclimatization period was applied at 25 °C prior to the feeding trial. The five treatments in triplicate were arranged in rectangular glass aquaria of 40 L in a completely randomized design with 10 zebrafish juveniles (5–5 male and female) in each. The initial individual wet body weight was 0.260 ± 0.035 g, and size homogeneity was tested by ANOVA where no significant difference (*p* > 0.05) occurred among the treatments. Aquaria were filled up with aerated tap water; thus, the oxygen concentration was maintained at 100% during the 14 days of feeding trial. A 16–8 h (light-dark) of illumination was provided. Each aquarium was filtered individually, and the flowing as well as aeration of water was provided by piped filters.

The accumulation process of *D. pulex* was replicated daily; thus, zebrafish juveniles were fed freshly enriched zooplankton the same time every morning during the trial without additional supplementation. The amount of zooplankton introduced into the tanks was adjusted to obtain complete consumption. A 50% of water exchange took place daily as well as aquaria were checked for dead individuals.

After the 14 days of enrichment period *D. rerio* individuals were collected by fish net and were rinsed with ultrapure water to reduce the positive error in the analytical results. The individual wet body weight was measured, and samples were kept frozen prior to the sample preparation. The sacrificed procedure was by physical methods suggested in the AVMA Guidelines on Euthanasia for fish reported by the American Veterinary Medical Association [51].

### Sample Preparation and Elemental Analysis

Samples were dried at 105 °C for 24 h in a drying cabinet until constant weight, and the dry body weight of the samples was measured on analytical balance (Precisa 360 ES). They were digested on an electric hot plate with 6.0 ml 65% (m/m) nitric acid (reagent grade, Merck) and 2.0 ml 30% (m/m) hydrogen-peroxide (reagent grade, Merck) at 80 °C for 4 h. After digestion, samples were diluted with 1% (v/v) nitric acid (reagent grade, Merck and Milli-Q water) to a final volume of 12 ml in volume-calibrated test tubes.

Water samples from aquaria were collected every second day to check the concentration of Fe and Mn: centrifuge tubes of 10 ml (PP) with screw caps were used for sampling and 1 ml of cc. HNO_3_ was added to preserve until elemental analysis.

Elemental concentration was determined by microwave plasma atomic emission spectrometer (Agilent MP-AES 4200). Auto sampler (Agilent SPS4), Meinhard® type nebulizer and double-pass spray chamber were used as well as a five-point calibration procedure was applied (ICP VI, Merc). Certified reference material was used (ERM-BB422, fish muscle) to verify that the measured elemental concentrations are equal with the elemental levels of the examined organisms. The recoveries were within 10% of the certified values for the metals. The wavelengths and measuring parameters were chosen based on the suggestion of the instrument’s software (MP Expert).

### Data Evaluation

Weight gain (WG) percentage was calculated from the measured initial and final wet weight (*W*_*i*_ and *W*_*f*_, respectively) data of *D. rerio* individuals:

$$ \mathrm{WG}\ \left(\%\right)=\left({\mathrm{W}}_{\mathrm{f}}-{\mathrm{W}}_{\mathrm{i}}\right)/{\mathrm{W}}_{\mathrm{i}}\cdotp 100 $$

Specific growth rate (SGR) was applied to describe the growing performance of *D. rerio* according to the following formula:

$$ \mathrm{SGR}\ \left(\%/\mathrm{day}\right)=\left({\mathrm{lnW}}_{\mathrm{f}}-{\mathrm{lnW}}_{\mathrm{i}}\right)/\mathrm{t}\cdotp 100, $$

where *W*_*f*_ is the final wet body weight and *W*_*i*_ is the initial wet body weight of the zebrafish individuals [52].

The bioconcentration factor (BCF) for *D. pulex* was calculated by dividing the Fe and Mn concentration measured in the zooplankton (*C*_*tissue*_, mg kg^−1^ dry weight) by the same concentration values of the rearing water media (*C*_*water*_, mg L^−1^) [28]:

$$ \mathrm{BCF}={C}_{tissue}/{C}_{water} $$

Trophic transfer factor (TTF) was calculated as the ratio of the tissue concentration of Fe and Mn measured in *D. rerio* (*C*_*predator*_) and the tissue concentration of the two elements measured in *D. pulex* (*C*_*prey*_) both given in mg kg^−1^ dry weight [53]:

$$ \mathrm{TTF}={C}_{predator}/{C}_{prey} $$

The statistical evaluation of experimental data was carried out in SpSS/PC+ software package. ANOVA was applied to study the WG, SGR, BCF, TTF, and elemental concentration results of the applied treatments. The homogeneity of variance was checked by Levene test, and significant differences were investigated by Tukey multi-comparison test where difference was considered to be statistically proven when *p* < 0.05. Redundancy analysis (RDA) was performed in Canoco for Windows 4.5 to study the interaction between the studied species and their environmental background: *D. pulex*/water and *D. rerio*/*D. pulex*, respectively.

## Results

### Survival and Growth Performance of *Danio rerio* Fed with Fe- and Mn-Contaminated Daphnia

**Fig. 1:**
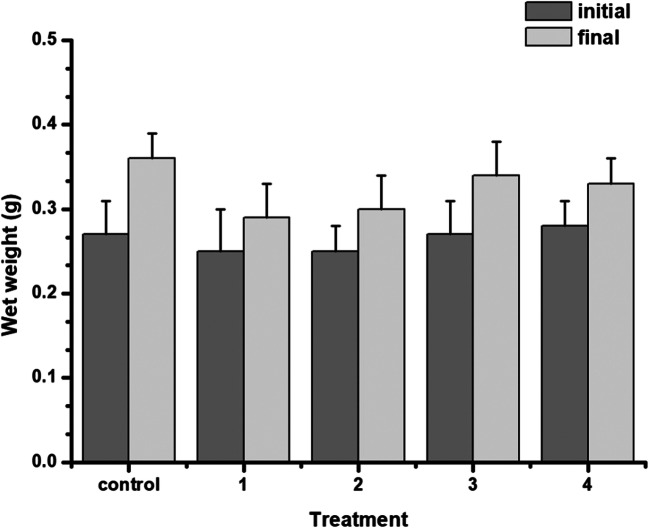
The average initial and final wet weight of zebrafish individuals in the different treatments (mean ± SE, *n* = 3) Control: fed by non-supplemented *Daphnia*, 1: fed by *Daphnia* supplemented with 5.70 mg L^−1^ Fe and 2.90 mg L^−1^ Mn, 2: fed by *Daphnia* supplemented with 5.70 mg L^−1^ Fe and 6.25 mg L^−1^ Mn, 3: fed by *Daphnia* supplemented with 15.0 mg L^−1^ Fe and 2.90 mg L^−1^ Mn, 4: fed by *Daphnia* supplemented with 15.0 mg L^−1^ Fe and 6.25 mg L^−1^ Mn

The survival of zebrafish juveniles was 100% in all treatments; thus, the applied concentrations of Fe and Mn did not cause the mortality of fish individuals during the experimental period. The average final wet weight of fish increased in all treatments compared to the initial values (Fig. 1) indicating that the metal supplementation did not result in growth abnormality; however, reduced growth performance was observed. The highest percentage values of SGR and WG were achieved by the control group (Table 1), which suggests that feeding Fe- and Mn-enriched *D. pulex* might had a negative influence on fish growth.

**Table 1:** Specific growth rate and weight gain percentage of *Danio rerio* in different treatments (mean ± SE, *n* = 3). Control: fed by non-supplemented *Daphnia*, 1: fed by *Daphnia* supplemented with 5.70 mg L^−1^ Fe and 2.90 mg L^−1^ Mn, 2: fed by *Daphnia* supplemented with 5.70 mg L^−1^ Fe and 6.25 mg L^−1^ Mn, 3: fed by *Daphnia* supplemented with 15.0 mg L^−1^ Fe and 2.90 mg L^−1^ Mn, 4: fed by *Daphnia* supplemented with 15.0 mg L^−1^ Fe and 6.25 mg L^−1^ Mn. Letters in lowercase indicate significant differences (*p* < 0.05)

Treatments	SGR (%/day) ± SE	WG (%) ± SE
Control	2.20 ± 0.63a	37.0 ± 11.7a
1	1.15 ± 0.24b	17.5 ± 3.93b
2	1.42 ± 0.47c	22.6 ± 7.96bc
3	1.68 ± 0.52c	27.3 ± 9.01c
4	1.15 ± 0.26b	17.6 ± 4.25b

### Elemental Concentration of Fe- and Mn-Enriched *Daphnia pulex* and *Danio rerio*

**Fig. 2:**
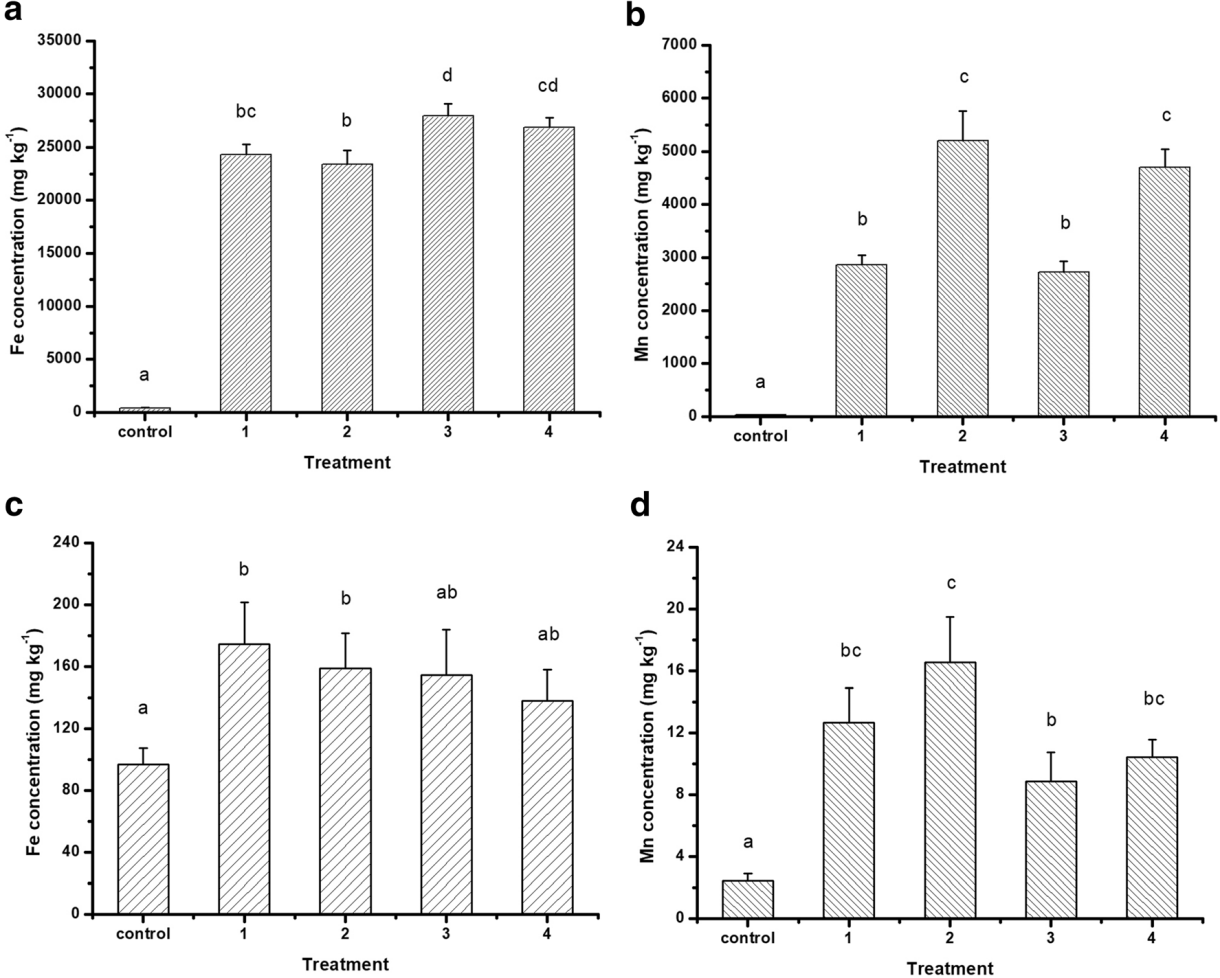
The Fe (**a**) and Mn (**b**) concentration of supplemented *Daphnia pulex* and zebrafish (**c** and **d**, respectively) (mean ± SE dry weight, *n* = 3) Control: non-supplemented, 1: supplemented with 5.70 mg L^−1^ Fe and 2.90 mg L^−1^ Mn, 2: supplemented with 5.70 mg L^−1^ Fe and 6.25 mg L^−1^ Mn, 3: supplemented with 15.0 mg L^−1^ Fe and 2.90 mg L^−1^ Mn, 4: supplemented with 15.0 mg L^−1^ Fe and 6.25 mg L^−1^ Mn. Letters above columns indicate significant differences (*p* < 0.05)

The Fe and Mn concentration of *D. pulex* calculated to dry weight is indicated in Fig. 2 a and b, respectively.

Both the Fe and Mn level of the zooplankton samples in all the applied treatments increased significantly compared to the control group (*p* < 0.001, *F* = 4.319 and *F* = 12.236, respectively). Considering the Mn content, the treatments containing the same Mn supplementation (2.90 mg L^−1^ in numbers 1 and 3 as well as 6.25 mg L^−1^ in numbers 2 and 4) did not differ statistically from each other (*p* > 0.05) (Fig. 2b). Similar results for Fe were gained (Fig. 2a): its concentration in treatment numbers 1 and 2 (both containing 5.70 mg L^−1^ Fe) is statistically comparable (*p* > 0.05) as well as in treatment numbers 3 and 4 (both containing 15.0 mg L^−1^) (*p* > 0.05). However, in contrast to Mn, the Fe level differed less between the treatments supplemented with the lower (5.70 mg L^−1^) and the higher (15.0 mg L^−1^) dosage (*p* < 0.05, respectively).

The average Fe and Mn concentration of zebrafish juveniles per treatments are indicated in Fig. 2 c and d, respectively. The Fe content of all groups increased compared to the control (*F* = 4.013, *p* < 0.05); however, the two different applied Fe concentrations did not affect the Fe level of the zebrafish individuals—no statistical difference was found either between treatments 1 and 2 (5.70 mg L^−1^) (*p* > 0.05) or between treatments 3 and 4 (15.0 mg L^−1^) (*p* > 0.05). The Mn concentration of the fish also increased significantly compared to the non-supplemented control group (*F* = 16.132, *p* < 0.001).

The elemental composition of *D. rerio* individuals can be seen in Table 2. No significant difference occurred in the composition of the samples regarding the trace element pattern either compared to the control (*p* > 0.05, respectively, and Ca: *F* = 1.668; Cu: *F* = 2.286; Mg: *F* = 1.271; Na: *F* = 0.895; Zn: *F* = 0.887) or between the treatments, except for Co (*p* < 0.01, *F* = 37.326).

**Table 2:** Elemental concentration (mg kg^−1^, dry weight) of *Danio rerio* in different treatments (mean ± SE, *n* = 3). Control: fed by non-supplemented *Daphnia*, 1: fed by *Daphnia* supplemented with 5.70 mg L^−1^ Fe and 2.90 mg L^−1^ Mn, 2: fed by *Daphnia* supplemented with 5.70 mg L^−1^ Fe and 6.25 mg L^−1^ Mn, 3: fed by *Daphnia* supplemented with 15.0 mg L^−1^ Fe and 2.90 mg L^−1^ Mn, 4: fed by *Daphnia* supplemented with 15.0 mg L^−1^ Fe and 6.25 mg L^−1^ Mn. Letters in lowercase indicate significant differences (*p* < 0.05)

Treatment		Ca (mg kg^−1^)	Co (mg kg^−1^)	Cu (mg kg^−1^)	Mg (mg kg^−1^)	Na (mg kg^−1^)	Zn (mg kg^−1^)
Control *N* = 30	Mean ± SE	29.346	0.346a	7.52	1046	2438	228
		1864	0.0555	0.608	44.5	85.1	15.1
1 N = 30	Mean ± SE	30.968	0.607b	8.63	1081	2449	245
		2854	0.118	1.16	84.7	277	24.3
2 N = 30	Mean ± SE	33.789	0.924c	9.75	1168	2637	255
		3754	0.101	1.32	69.3	129	14.7
3 N = 30	Mean ± SE	32.963	1.06c	7.99	1094	2369	244
		2073	0.0623	0.715	52.8	252	11.4
4 N = 30	Mean ± SE	33.714	1.09c	7.97	1108	2462	255
		2518	0.0842	0.716	65.8	187	19.4

### Interaction Between the Fe and Mn Levels of Water, *Daphnia pulex*, and *Danio rerio*

**Fig. 3:**
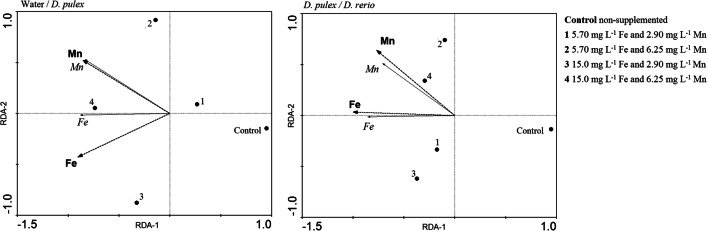
The RDA biplots of the Fe and Mn level of rearing water and *D. pulex* as well as of *D. pulex* and *D. rerio* (solid arrow: elemental concentration of water/*D. pulex*, dashed arrow: elemental concentration of *D. pulex*/*D. rerio*. Filled circles with numbers indicate experimental groups)

Redundancy analysis was applied to assess the interaction between the level of Fe and Mn in the treatments and in the studied aquatic species, which is a commonly applied statistical technique to explain and model different cause–effect relationships [54]. The RDA biplot regarding the Fe and Mn concentration of the rearing water and *D. pulex* is indicated in Fig. 3. In the first component (RDA1) the correlation between Fe and Mn concentration of the rearing media and the concentration of the same elements in *D. pulex* was 0.884, while in the second component (RDA2) the correlation was 0.858. The cumulative percentage variance of elemental concentration of the zooplankton was 77.2 (RDA1) and 78.1 (RDA2). The relation regarding the species–environment connection revealed to be 98.9 (RDA1) and 100.0 (RDA2). The biplot shows a relatively strong correlation between the Fe levels of the Fe- and Mn-contaminated water as well as the reared zooplankton individuals, and a total agreement between the Mn concentrations.

Very similar results were obtained for the RDA of the Fe and Mn content of *D. pulex* reared in contaminated water media and *D. rerio* fed by the enriched zooplankton organism, shown also in Fig. 3. The correlation between Fe and Mn concentrations in the relation of *D. pulex* and *D. rerio* in RDA1 was 0.855, while in RDA2 the correlation was 0.987. The cumulative percentage variances of Fe and Mn level of the zebrafish were 72.4 (RDA1) and 73.3 (RDA2). The species–environment connection was found to be 98.8 (RDA1) and 100.0 (RDA2).

### Bioconcentration and Trophic Transfer Factor of *Daphnia pulex* and *Danio rerio*

Bioconcentration factors calculated from water to zooplankton for Fe and Mn are summarized in Table 3. Statistically significant difference occurred (*p* < 0.05, Fe: *F* = 21.727; Mn: *F* = 3.407) between the treatments containing 5.7 mg L^−1^ Fe (treatments 1 and 2) and the treatments containing 15.0 mg L^−1^ (treatments 3 and 4). Groups supplemented with the same dosage of Fe did not differ from each other significantly (*p* > 0.05). Higher supplemented level resulted in lower BCF values. The same is true for Mn: the higher dose of enrichment provided lower BCF proving the relation between the measured concentration of the two elements applied in the treatments and in the zooplankton organisms. The BCF data for Fe in the treatments 1 and 2 are nearly four times to that of the Mn. The TTF values are summarized in Table 4 for *D. rerio* exposed to dietary Fe and Mn via *D. pulex*, showing less difference yet still statistically proven (*p* < 0.05, Fe = *F* = 2.597; Mn: *F* = 5.092).

**Table 3:** Bioconcentration factors (mean ± SE) of Fe and Mn from water to *Daphnia pulex*. Control: non-supplemented, 1: supplemented with 5.70 mg L^−1^ Fe and 2.90 mg L^−1^ Mn, 2: supplemented with 5.70 mg L^−1^ Fe and 6.25 mg L^−1^ Mn, 3: supplemented with 15.0 mg L^−1^ Fe and 2.90 mg L^−1^ Mn, 4: supplemented with 15.0 mg L^−1^ Fe and 6.25 mg L^−1^ Mn. Letters in lowercase indicate significant differences (*p* < 0.05)

Elements	Treatments	BCF
Fe	1	4591 ± 105a
	2	4371 ± 169a
	3	1902 ± 31.1b
	4	1864 ± 33.0b
Mn	1	1044 ± 54.3a
	2	925 ± 96.3b
	3	1006 ± 35.9a
	4	799 ± 17.3b

**Table 4:** Trophic transfer factors (mean ± SE) for Fe and Mn from *Daphnia pulex* to *Danio rerio*. Control: fed by non-supplemented *Daphnia*, 1: fed by *Daphnia* supplemented with 5.70 mg L^−1^ Fe and 2.90 mg L^−1^ Mn, 2: fed by *Daphnia* supplemented with 5.70 mg L^−1^ Fe and 6.25 mg L^−1^ Mn, 3: fed by *Daphnia* supplemented with 15.0 mg L^−1^ Fe and 2.90 mg L^−1^ Mn, 4: fed by *Daphnia* supplemented with 15.0 mg L^−1^ Fe and 6.25 mg L^−1^ Mn. Letters in lowercase indicate significant differences (*p* < 0.05)

Elements	Treatments	TTF × 10^−3^
Fe	1	7.19 ± 0.410a
	2	6.74 ± 0.627a
	3	5.51 ± 0.616b
	4	5.15 ± 0.720b
Mn	1	4.45 ± 0.208a
	2	3.26 ± 0.555b
	3	3.26 ± 0.410b
	4	2.23 ± 0.351c

## Discussion

The accumulation tendency of different pollutants carries essential information regarding the water quality, the well-being of aquatic ecosystems, and is directly related to human health. The appearance of iron and manganese in water ecosystems can originate from industrial and agricultural emissions; however, the natural geological background can result in elevated levels.

The growth performance of zebrafish juveniles was found to be slightly affected by the environmentally toxic levels of Fe and Mn since lower values of SGR and WG were detected compared to the control group. This finding is in contrast with our previous experiment where the sublethal level of Fe and Mn retention was studied from water via the gills of *Common carp* (*Cyprinus carpio*) [28]. In that study ten times lower level of the two elements were applied in a very similar experimental setup; thus, we can conclude that the elevated dose of Fe and Mn supplementation in present investigation reached the value where it affects the growth performance. The negative influence of Mn in fish is mainly due to the increased oxidative stress it provokes [55–58]. Gabriel et al. described the toxicity of Mn exposed to juvenile *Colossoma macropomum* by evaluating oxidative stress parameters and found that biomarkers changed significantly in the tissues of the studied fish individuals proving specific toxicity of Mn to the different organs [58]. In contrast, low level of Mn supplementation in fish diet can have opposite effect by promoting growth and normal skeleton development [20].

The Mn level in the zooplankton organisms increased in parallel with the dose of the applied supplementation. The same phenomenon was observed by Fehér et al. in a 24-h enrichment trial of *Artemia nauplii* with MnCl_2_ [52]. The high bioconcentration potential of mainly herbivores *Daphnia* species is published compared to omnivorous species, such as rotifers, for Cd, As, and Pb [33]. However, the two different applied Fe concentrations in treatments did not result in significantly different uptake level of the zebrafish individuals. This finding correlates with the results obtained by Harangi et al., who conduct experiments to study the accumulation of metals in juvenile carp exposed to sublethal levels of Fe and Mn [28]. Either the smaller accumulation tendency of Fe can be one of the possible explanations or the higher hydrolyzation potential of Fe in the model media in which the zooplankton organism was reared. However, compared to the control group, the concentration of both the studied elements increased. This finding proves that nevertheless the main uptake path for dissolved Fe and Mn for fish is via the gills; retention is also efficient through the digestive track. Since the level of the other essential elements did not change upon the Fe and Mn treatments, we can further conclude that the applied concentration of Fe and Mn did not affect the metabolism process of other metals.

The good correlation indicated in the redundancy analysis proves the direct connection between the Fe and Mn concentration of the zooplankton organisms as well as the zebrafish individuals with the applied treatments, thus the ability of these elements to concentrate in the aquatic food chain. Fehér et al. used RDA to reveal the effect between the elemental content of a saltwater zooplankton (*Artemia naupli*) species and Barramundi (*Lates calcarifer*) larvae [52]. In their experimental setup the zooplankton was reared in a Mn, Zn, and Co supplemented water media and barramundi individuals were fed by the enriched *Artemia*. Similar to present study, they found correlation between the environmental and species concentration data for Mn.

Bioconcentration of organic and inorganic pollutants in aquatic organisms is a significant parameter to assess the effect of chemicals emitted to the environment. It can be expressed numerically in experiments conducted under laboratory conditions by the bioaccumulation factor, which can be used for regulatory purposes if certain limitations are considered [59]. The BCF of present study proved the relation between the level of Fe and Mn in the treatments and in the zooplankton organisms. Manganese is a commonly occurring element in the aquatic environment, in both underground and surface waters. Although it is an essential element, even a short-term waterborne exposure to higher concentration can indicate peroxidative damage in fish tissues [60]. Marins et al. in their fresh study from 2019 found that long-term exposure to Fe and Mn in a concentration commonly found in groundwater may cause damage to chromosome levels and changes in locomotor and exploratory behaviors of adult zebrafish [61]. It was further concluded by Tu H. et al. that developmental exposure to Mn along with Cd and Pb statistically reduced the velocity and distance of the larval swim of zebrafish [62]. Altenhofen et al. studied the effect of MnCl_2_ exposure on cognition and exploratory behavior in adult and larval zebrafish. Both adults and larvae reacted with decreased distance traveled and absolute body turn angle as well as increased apoptotic markers were found in their nervous system. The research group concluded that the prolonged Mn exposure resulted in locomotor deficits that can damage the dopaminergic system [63].

The BCF results for Fe in the first two treatments of present study are higher than the same data for Mn. In our previous study the Fe and Mn bioconcentration was investigated from water to fish (*Common carp*) in a model experiment and similar tendency was found [28]. Voigt et al. also reported much higher BCF for Fe compared to Mn in the tissue of *Geophagus brasiliensis* originating from Alagados Reservoir, Ponta Grossa [64]. Iron can affect both directly and indirectly the living organisms in aquatic ecosystems and, according to the suggestion to Vuori, the ecotoxicological aspects of Fe should be further analyzed with organisms of different levels [65].

Liu et al. in their paper suggested the further investigation of the relative importance between the aqueous and dietary exposure of metals to fish in order to deeper understand the processes of metal transfer into the fish body [30]. In agreement with this conclusion in our last study we investigated the effect of waterborne Fe and Mn on the tissue of indicator fish, while in present work the dietary Fe and Mn is tested. Trophic transfer factor can be used for the latter purpose to express the bioconcentration from zooplankton to fish [53]. The results of TTF are more homogenous compared to BCF proving less difference between the applied concentration of dietary Fe and Mn. The values suggest that transfer definitely occurs from zooplankton to fish for the studied metals yet in a lower extent than directly from water to fish, as described in our previous study [28]. According to the results of Zhu et al. *D. rerio* could also accumulate nTiO_2_ by aqueous exposure with higher bioaccumulation factors compared to the dietary intake.

Biomagnification is not observed in present study along the water to *D. pulex* to *D. rerio* trophic route. This finding meets previously published research data stating that BCF and TTF usually increase along the given aquatic food chain right until the level of fish where it starts to decrease significantly. Pianpian et al. concluded in their meta-analysis that the average MeHg bioaccumulation from water to either seston or zooplankton was over 6 order of magnitude greater than the biomagnification from zooplankton to preyfish [66]. Mathews et al. studied the retention of MeHg, Cd, and Po in an estuarine food chain and found a greater biomagnification at the trophic step of *D. pulex* feeding on phytoplankton [67]. This phenomenon is commonly explained by the higher availability of toxic elements to organisms at lower trophic levels [68]. Our study further confirms Oweson and Hernroth finding that Mn tends to accumulate strongly in aquatic organisms situated lower in the food web [69].

It was also long found that aquatic vertebrates as fish can efficiently excrete ingested heavy metals, especially the no-essential and toxic ones [70]. Increased BCF factor for Fe and Mn was observed from water to fish in our previous experiment compared to the current one, where only in the absorption trough the respiratory and dermal surfaces was considered [28]. It further indicates that retention of waterborne Fe and Mn is stronger compared to the dietary one; however, the accumulation extent of elements vary among the metals and metal species showing very different behavioral patterns. For example, the main route of Cd accumulation in marine environment is considered to be the dietary exposure as well as most of the other metals that have been studied in the past decades [71]. For freshwater ecosystems Cd was found to enrich strongly in the trophic chain, while no enrichment tendency of Cu was observed in the same survey [72]. The accumulation tendency of Mn was investigated by Niemiec et al. in a field study resulting in much higher BCF values from water to *Ciprinus carpio* in the analyzed ecosystem than other routes.

It is also proven by several field studies and laboratory experiments that toxic elements are accumulated in certain organs contributing to a small extent to the total weight of the fish body explaining the lower level of retention results. This finding is confirmed for waterborne Fe and Mn in our last experiment where both were absorbed in the highest level in the liver of *Common carp* juveniles [28]. Metals stored in the form of granules usually have lower bioavailability to the predator organisms [73].

The so-called grow dilution may also take part in the smaller TTF values from zooplankton to *D. rerio* compared to the BCF data given for water to *D. pulex*. This pseudo-elimination process occurs due to the increase of fish tissue during the experimental time and results in a lower determined BCF values even more under laboratory circumstances [59, 74].

## Conclusion

Present work indicates the importance of model experiments to gain more detailed information regarding the accumulation tendency of metals, not limited solely to the non-essential ones. It highlights that laboratory circumstances provide the possibility to investigate only one absorption pathway at a time and to selectively exclude the others. Iron and manganese are essential elements to a certain level above which both have negative influence on aquatic organisms. Our work proves that the main step from the point of bioconcentration occurs in the lower part of the trophic system since higher accumulation tendency was observed in the first stage of the investigated food web. The results demonstrate that concentrations of Fe and Mn well above the environmentally accepted levels still do not cause mortality neither biomagnification in the studied organisms; however, retention in the growth performance of *D. rerio* was observed. The lack of acute toxicity thus does not translate into no negative effect. It is mentioned in literature that damage to chromosome levels and changes in locomotor and exploratory behavior is resulted in long-term exposures. Further model studies are therefore recommended to investigate the dietary Fe and Mn absorption in the different organs and tissues of fish to supplement research approaches determining the biochemical adverse effects, such as oxidative stress.
